# Notes on Self-Poisoning and Its Treatment, with Special Reference to Some Physical Methods

**Published:** 1896-12

**Authors:** C. J. Whitby


### NOTES ON SELF-POISONING AND ITS TREATMENT, WITH SPECIAL REFERENCE TO SOME PHYSICAL METHODS.

BY

C. J. Whitby, B.A., M.D. Cantab.

Professor Bouchard says that "the organism, in its normal, as in its pathological state, is a receptacle and a laboratory of poisons."[1] And Dr. Oliver, in the preface to his translation of Bouchard's lectures, states that every moment of our lives we run the risk of being overpowered by such poisons. In this paper I have brought together some facts of clinical significance gleaned from the recent literature of this important subject, and especially from the above-named lectures.

An important place on the list of those poisons to the ill-effects of which the organism may, by the failure of elimination, be at any time subjected belongs to the mineral constituents, and especially to the potassium compounds, of the food.[2] For potassium is a powerful convulsive. Of the various salts constantly entering the circulation a considerable portion is retained by the blood-globules, which have a marked affinity for potassium, and the part so retained may be liberated in the plasma by any cause which induces the death and disintegration of these globules. Minerals are of course constantly being

[1] *Lectures on Auto-Intoxication in Disease.* By Ch. Bouchard. Tr. by Thomas Oliver, M.D., 1894, p. 14.

[2] *Op. cit.*, p. 10.

eliminated by the healthy kidneys, and Bouchard attributes one-third of the normal toxicity of urine to their presence. One result, therefore, of the failure of renal excretion will presumably be the manifestations of mineral poisoning, and especially the convulsive effect of potassium.

Toxic properties may be alleged of most, if not all, the secretions and excretions normally present in the alimentary canal, or, indeed, elsewhere. Thus a toxin chemically characterised by strong reducing powers may be extracted from fresh saliva. The toxicity of bile is a subject of more practical importance. Bouchard asserts[1] that during twenty-four hours a man makes by the activity of his liver alone enough poison to kill three men of his own weight, 1 kilogramme of bile yielding enough to kill more than 2,800 grammes of living tissue. Man forms in eight hours by his hepatic secretion alone enough poison to kill himself. Bile is, volume for volume, nine times as poisonous as urine; in an equal period the biliary secretion represents a toxic power six times that of the urinary secretion. Of the biliary ingredients bilirubin has by far the greatest toxic power, being ten times as poisonous as either of the bile-salts, and constituting by its presence two-thirds of the total toxicity of the secretion. The poisonous colouring matter of bile on contact with the acid chyme is, as a rule, suddenly precipitated in the small intestine, and by a somewhat similar process the biliary salts and acids are also rendered insoluble. This change retards and limits reabsorption, and so diminishes the risk of self-poisoning. Moreover, the liver, as Schiff has demonstrated, arrests the reabsorbed biliary ingredients and restores them to the intestines or transforms them into harmless matter. This protective agency of the liver is by no means confined to the arrest or modification of the biliary toxins, and as a safeguard against self-poisoning is only second to the rôle of the kidneys. Thus the alkaloid pepto-toxin which results from the peptic digestion of fibrin is an extremely poisonous base, and the normal products of digestion when introduced (as by injection into the jugular vein) direct into the general circulation, so as to escape the synthetic activity of the liver,

[1] *Op. cit.*, p. 224.

may cause a fatal toxæmia in the animal so treated.[1] Gautier has established the existence of a whole class of alkaloids (termed leucomaines and of "great importance . . . in the genesis of disease") which, like pepto-toxin, are necessary products of vital functions. To this class belong some at least of those toxic products of cellular metabolism which, Bouchard asserts, are constantly being absorbed by lymphatics and vessels from the extra-cellular fluids of the body.

Returning to the poisons of gastric or intestinal source, we note that although the occurrence of potentially deleterious products is a necessary result of normal digestion, their quantity and virulence will be greatly augmented by conditions tending to acid or putrid fermentation. Omitting, as beyond our present scope the consideration of poisoning by the ingestion of putrid or putrefying foods, it is worth remembering that simple apepsia may be the means of producing a condition that is practically identical. For in cases where even fresh and wholesome food, through debility or idiosyncrasy, fails to evoke the proper digestive response, the hydrochloric acid (which normally acts as an antiseptic) being absent, putrefaction of the food occurs and toxic symptoms ensue. Hence, presumably, the faintness, mental oppression, etc., which in dyspeptics follow a full meal. Toxic alkaloids are (as their nitrogenous basis would suggest) most conspicuous as results of the putrefaction of *proteid* substances, and it is noteworthy that the final products of such decomposition (such as ammonias and organic acids) diffuse readily and are readily absorbed.

An important predisposing cause of chronic auto-intoxication is gastric dilatation. Bouchard[2] believes this condition to be very common, and asserts that some degree of it may be found in seven out of eight cases of chronic dyspepsia. In many such cases he has observed certain nodosities which he considers pathognomonic of gastrectasis, and attributes to the effect of absorbed acetic acid. Their favourite site is the second phalangeal articulations of the hands, where they are due to

[1] *Ptomaines and other Animal Alkaloids.* By A. C. Farquharson, M.D., 1892, p. 102.

[2] *Op. cit.*, pp. 165–6, 174.

an enlargement of the bases of the second phalanges, but they may affect other joints. They somewhat resemble the rheumatic nodules of Heberden (which, however, affect the third phalangeal articulations), and are also comparable to the beaded ribs of rachitis.

Seeing that toxins are commonly so abundant in the stomach and intestines, it is somewhat surprising how rarely toxic accidents occur. Possibly some poisons are (as Stich suggests) neutralised, as by dialysis, in process of absorption, or in the circulation by the activity of leucocytes. "But if," says Bouchard, "we cannot demonstrate that these poisons enter the blood, we can prove that some of them leave it (and practically unchanged) in the urine." There is, moreover, a quantitative and qualitative parallelism between the alkaloids present at a given time in the fæces and those which are being eliminated in the urine. If, by antisepsis or by the administration of charcoal, we suppress intestinal fermentation or neutralise its products, we diminish in proportion the toxicity of the urine. Phenol is, almost certainly, eliminated unchanged, and the urinary percentage of indican (a derivative of indol) rises or falls with the increase or diminution of indol in the fæces.

Probably (as was suggested above) some of the absorbed gastric and intestinal toxins are modified in passage through the liver. Heeger has injected into the portal vein blood containing nicotine, morphine, and other alkaloids. The blood coming from the liver contained less of these.[1] The experiments of Schiff and G. H. Roger practically demonstrate that the liver not merely arrests poisons but actually destroys them. The alcoholic extract of rotten meat injected into the portal vein is twice less toxic than when introduced into the general circulation.[2] Hence, probably, the great difference in the potency of oral and hypodermic medication.

Remembering that the manufacture of leucomaines is an invariable result of normal cellular metabolism, it is not surprising that the liquor sanguinis which receives them has an average toxicity of measurable degree. Bouchard calculates that a kilogramme of living blood contains in its plasma alone

[1] Bouchard, *op. cit.*, p. 17. [2] *Op. cit.*, pp. 142–3.

enough poison to kill more than 1,250 grammes of living matter,[1] and that a man would die toxæmic if his blood contained ten times its normal quantity of poison. Cellular metabolism is to a certain extent independent of the presence of free oxygen, and it is probable that those substances which are most poisonous are especially the waste products of such anaërobiotic activity. Conversely, by the inhalation of oxygen the toxic products of cell metabolism are diminished, and those actually circulating in the blood are rendered less virulent and easier of elimination. And here we may also note in passing the importance of the respiratory function as a means of excretion, and especially the excretion of such volatile nitrogenous products of anaërobiotic metabolism as have just been alluded to. Du Bois-Reymond "terms the organic poison excreted . . . 'anthropotoxin,'"[2] and to it is due the peculiar stuffy odour of the air of a room which has been breathed in for some time. Besides poisons of normal or quasi-normal occurrence, there are cases in which the vitiated elaboration of matter by the system results in the production of *anomalous* toxins. One such is acetone, the cause of acetonæmia, which occurs in the blood and urine of the subjects of diabetic coma, and gives a claret colour with perchloride of iron.[3] Certain of the symptoms of cholera are due to an anomalous poison elaborated either by the microbes or by the system itself as influenced by them.[4]

There can be no question but that the principal safeguard against self-poisoning is the functional integrity of the kidneys.[5] Since normal urine is poisonous in a high degree, the importance of renal permeability is obvious. The toxicity of healthy urine varies considerably according to cerebral or muscular activity, sleep, diet, etc.; but, on the average, a man excretes in two days and four hours enough urine to poison himself.[6] Night urines are, upon the whole, less toxic than those of the day. The toxicity of both is greatly reduced by muscular exertion on the part of the subject, or by his exposure to compressed oxygen. The urines of sleep are always markedly convulsive, while those of the day period are but slightly so, but produce narcosis. It

[1] *Op. cit.*, p. 76. [2] Farquharson, *op. cit.*, p. 122.
[3] Bouchard, *op. cit.*, pp. 142, 247, 287. [4] *Op. cit.*, p. 265. [5] *Op. cit.*, p. 287.
[6] *Op. cit.*, p. 35.

would seem that during the day the body forms, and in part eliminates, a substance which in accumulation would produce sleep, and during the night a convulsive agent which would induce waking.[1] Normal urine contains at least seven poisons: a diuretic substance (urea), a narcotic, a salivating principle, one that contracts the pupil, one that lowers the temperature, and two convulsives—the one mineral, the other organic.[2] Of the total toxicity Bouchard assigns two-fifths to the colouring matters, etc., separable by charcoal; one-third to minerals, especially salts of potassium; about one-seventh to urea, and a small fraction to ammonia.[3] In febrile urines the pigments are ten to twenty times more abundant than in normal urines, while the potassium (liberated by cellular disintegration) is doubled or trebled. Hence, rather than to the presence of any *special* poison, is due the great convulsive power of such pathological urines. The urine in cases of albuminuria is often of low toxicity, while on the supervention of uræmia, if suppression does not occur, its toxicity practically disappears. Uræmia is to be regarded as a mixed poisoning, not by urine, but by accumulation of substances which should have become urine, the sources and natures of which have just been described. The immediate cause of suppression of urine may be a deficiency of urea, the physiological diuretic. A characteristic example of self-poisoning (as all its symptoms from the early pupillary contraction to the last convulsive throe attest) is the ordinary death-struggle, the onset of which is likewise often determined by renal failure.

From what we know of its causation, some general rules for the prevention and treatment of self-poisoning may be deduced. Thus, by careful dieting (to take for granted more obvious, but all-important, hygienic precautions) we can minimise first the introduction of toxins and secondly their generation by putrid or acid fermentation. Or we can arrest such fermentation by the use of antiseptics, or fix and prevent the absorption of its products with charcoal. By stimulation of the liver we can perhaps assist that organ in arresting and destroying absorbed poisons. Failing this, we must promote elimination, by the

[1] *Op. cit.*, p. 41. [2] *Op. cit.*, pp. 60–5. [3] *Op. cit.*, p. 120.

skin, lungs, bowels, and, above all, the kidneys, the chief natural route of toxic egress. Copious drinks, cold baths and cold enemata are the means Bouchard recommends to this end; and in cases where the absorption of putrid products is to be feared milk has the advantage of inducing solid and scanty motions, while it contains little potassium and but few extractives. Diaphoresis is often useful (especially, I think, in chronic cases), but we must be careful not to unduly diminish the amount of the renal excretion. In certain cases we may hope to counteract by physiological antidotes, or possibly by the administration of oxygen gas, the effects of toxins in the blood. In convulsive cases the use of sodium-salts is preferable to that of salts of potassium ; and broths rich in minerals and extractives are to be avoided. In acute cases a critical period may be tided over by the aid of stimulants.

Anyone who dispassionately considers the facts which have just been reviewed cannot but suspect that some kind or degree of self-poisoning is the fundamental factor of many apparently very diverse diseases. Consider for a moment the extreme frequency of the following conditions; namely, excess of food rich in nitrogenous materials, minerals, or extractives; excess of easily fermentable or putrefiable foods; dyspepsia, apepsia, and gastric dilatation; pre-occupation and consequent partial and temporary disablement of the liver, due to an excess of fatty food, alcoholic indulgence, or other dietetic error,—or, if more habitual, to chronic intemperance, a sedentary habit, or the occupation of an ill-ventilated office or dwelling; limitation of the respiratory and excretory activity of the lungs and skin; functional or organic deficiency on the part of the kidneys. It is a safe assumption that the usual result of any one of these more than common conditions will be at least a temporary rise in the toxicity of the blood; consequently, that self-poisoning of some kind or degree plays a part in determining the symptoms of almost every complex pathological condition. For if an excess of food be taken, some of the products of its decomposition will probably escape the synthetic activity of the liver and pass through from the portal into the general circulation. If, on the other hand, the toxic by-products of digestion be of only normal quantity and virulence, but if, on reaching the liver, they find that organ temporarily disabled, the result will be practically the same. Again, supposing the poisonous products which from all sources enter the circulation to be only of normal quantity and power, but the renal function temporarily or permanently deficient, the inevitable consequence (unless or until some other outlet be provided) can but be a relative toxæmia.

The "other outlet" of which one immediately thinks in this connection is, of course, the skin, whose myriad sweat-glands Nature would seem to have provided as a natural safeguard against the perils of renal insufficiency. That cutaneous elimination may be regarded as an auxiliary or even an alternative to renal excretion is in practice generally admitted. Witness the classical treatment of acute nephritis by diaphoresis. If urea and uric acid are not (as has nevertheless been claimed by some observers) normal constituents of sweat, they have, at any rate, been often detected in it in considerable quantities in pathological conditions. So also have blood, albumen, oxalate of lime, sugar, lactic acid, indigo, bilirubin and other pigments. It is a matter of common knowledge that drugs, such as iodine, the iodides and tartaric acid, are eliminated by the skin. Probably then the chemical qualities, as also the specific gravity, of the sweat vary like those of the urine in some as yet undetermined relation to the condition of the blood. Bouchard, who speaks[1] somewhat slightingly of diaphoresis as compared with diuresis, warns us not to unduly diminish renal excretion by stimulating cutaneous elimination. It is conceivable that there may be a physiological incompatibility between *medicinal* diaphoresis and diuresis, but the case is different as regards *thermal* diaphoresis which can quite well be combined with renal stimulation. In the Turkish bath, for instance, we have a therapeutic agent of the first order, and one which, in the wide class of cases under consideration, is of almost universal applicability. Water-drinking is part of the routine of a properly-administered Turkish bath, and pure water is one of the best of diuretics. And apart from this even, my own

[1] *Op. cit.*, pp. 276–7.

experiments, so far as they have gone, tend to the conviction that the activity of the kidneys is rather increased than diminished by the Turkish bath, the average volume of urine passed in the twelve hours following a bath being slightly greater than that passed on days when the same extra quantity of water was drunk at the same hour but no bath taken; while the average specific gravity was in both cases about the same. As regards the use of hot-air baths in cases of auto-intoxication, there is another theoretical consideration which tells in its favour—the fact, namely, that exposure in, and inhalation of, an atmosphere heated to from 150° to 250° F. may raise the temperature of the blood several degrees; and this rise, slight and temporary though it be, will favour the oxidation of leucomaines, etc., thereby rendering them comparatively innocuous. But justice will never be done to the value of the physical methods until a scientifically constructed hydro-thermal installation is considered as necessary as an electrical department to the equipment of a good general hospital.

In conclusion, I will quote some necessarily brief particulars of a few cases where physical treatment proved decidedly beneficial, cases which (however different in other respects) resemble one another nevertheless in the fact that self-poisoning of some kind or degree is pretty clearly indicated:

CASE 1.—A lady of twenty-seven came under my care just after her fourth attack of acute rheumatism. She was suffering from aortic stenosis of long standing, but her chief present complaint was of rather severe rheumatic pains in the shoulders, elbows, wrists, fingers, ankles and knees. There was also slight œdema of both ankles, and a systolic bruit of blowing character was audible at the base of the heart. In connection with this point I would mention the recently published experiments of Dr. Lazarus-Barlow, which warrant the conclusion (anticipated in part by Wooldridge in 1889) that œdema, even the cardiac variety, is never due to purely mechanical causes, but proximately to local disturbances of nutrition, and especially to the poisoning of the tissues by retained waste products.[1] In this case I further noted that the skin was dry and scaly, and scaly eczematous patches occupying the site of healed ulcers were present on the inner surface of the right leg and above the left internal malleolus. The patient was treated with mild Turkish baths of brief duration, followed by a simple ablution, also with massage and dry faradism to the painful parts. The pain and œdema rapidly subsided, the general health improved markedly, and she left within a month, practically well but for the valvular lesion.

[1] *Brit. M. J.*, 1895, i. 634, 691.

Case 2.—Mr. H., aged 61, a small thin man, looking very ill, complained of asthma and general weakness. Until six years before the date of his coming to me he had enjoyed fair health, but broke down then through malnutrition from dyspepsia, attributed by the patient in the first place to the neglect and consequent loss of nearly all his teeth. His weight had diminished from eleven to eight stone, his hands were purple from venous congestion and were cold to the touch. He suffered from prolapse of the rectum during defæcation, due apparently to simple relaxation of the parts involved, and he was generally in a miserably weak condition. Respiration was habitually wheezy, but there were also frequent nocturnal paroxysms of dyspnœa resembling true asthma. Auscultation revealed little beyond a certain amount of emphysema. He wore a fairly good set of artificial teeth, but still suffered greatly from indigestion. He was carefully dieted, the foods prescribed being chiefly of a light kind (such as fish or fowl and farinaceous materials) and ordered to drink milk freely, and to take four or five mild Turkish baths weekly, with other baths of less importance. The quantity of milk had soon to be reduced, because of constipation, to about a pint in the day, but the appetite improved wonderfully, and in three weeks he had regained five pounds of body-weight and the nocturnal asthma was immensely relieved. So great an improvement can hardly be accounted for as the result of mere diet and change of air. My own explanation would be that the asthma was kept up by morbific products due to incomplete digestion and deficient aëration, and that the vicious circle thus established was (for a time) broken by their elimination through the skin.[1]

Case 3.—Miss M., aged 24. This patient was sent to me by Dr. E. Jones, of Cardiff, who gave the following particulars of the case as noted by him twelve months earlier: "The patient complains much of abdominal distension; there is alternate polyuria and dysuria. Menstruation regular, lasts two days, and is painful throughout. The skin is dry, the bowels are costive, and she is somewhat anæmic. She complains also of palpitation and morbid cephalic sensations." Abdominal palpation revealed slight tenderness over the right ovary. The urine was free from albumen. The patient is a publican's daughter, and is a good deal in the bar. (On this point I questioned her closely, and she admitted taking beer, but in moderation only; and I saw no reason to disbelieve her.) When first seen by me a year later, the condition was much the same. There was a constant sense of abdominal distension, aggravated after food, and due in part at least to flatulency, for attacks of palpitation were frequent, and the tongue was large and coated with a thick yellow fur. The urine was alternately pale and copious or scanty and loaded with urates, the spirits were depressed, and although she slept heavily she awoke every morning feeling dull and unrefreshed. She complained that her ankles and eyelids swelled towards night, but œdema was not perceptible. In this case the existence of catarrhal gastritis, with fermentative decomposition of the ingesta and the absorption of toxic products with which the deranged liver was in no condition to deal effectually, was matter of clearest inference. The treatment consisted of Turkish baths, mustard fomentations to the hepatic region (liver-packs), and a suitable diet. Hot water to be drunk freely. The patient made an excellent recovery within the month of her stay, and two months later I learnt that the improvement had been more than maintained.

[1] Bouchard, *op. cit.*, pp. 164, 171.

CASE 4.—J. M. H., aged 35. Malarial cachexia, lithiasis. The patient, a seafaring man, sent to me by Dr. Ogilvie, had been employed on long service in and about New Guinea. During the whole period of this service he had suffered from severe but irregular attacks of malarial fever, but especially during the last two years, when he had been much on land. He had recently been jaundiced, and the urine was still very dark. On examination, I found it free from blood and albumen, but of high specific gravity, and loaded with pink urates. The liver and spleen were much enlarged, and there was hypertrophy of the left ventricle. The pulse was 104, full and soft, and the temperature normal. He was taking quinine and arsenic, but the quantity was diminished while under my care. The complexion was sallow, but anæmia was not extreme. The treatment consisted of Turkish baths, mustard body-packs, copious draughts of hot water, and a suitable diet. He could only remain a fortnight, but on his return Dr. Ogilvie found the urine normal and the general health greatly improved. He also reported a slight reduction in the size of the liver and spleen.

It would be easy to multiply examples, but these four cases will suffice to illustrate the certainty and ease with which benefit is afforded by simple treatment on these lines in cachexial conditions of various kinds. The treatment is preventive as well as curative, because (with the exception perhaps of an occasional purgative) medicines are usually withheld, so that the patient's attention is naturally directed to the importance of care and moderation in the matter of food and drink, the need of fresh air, exercise, and so forth. So long as drugs are being taken there is a noticeable tendency to rely exclusively upon them for relief. In prescribing Turkish baths it is necessary to be explicit as to the details of temperature and duration, and in cases of debility the shampooing is often better omitted. Twenty to thirty minutes' exposure to a temperature of 125° F., followed by a simple spray bath, or even a tepid sponging, may be regarded as a minimum "dose."

